# De Novo Heterozygous Mutation in FGFR2 Causing Type II Pfeiffer Syndrome

**DOI:** 10.1155/2022/4791082

**Published:** 2022-06-28

**Authors:** Rafat Mosalli, Alfia Fatma, Mohammed A. Almatrafi, Mayada Mazroua, Bosco Paes

**Affiliations:** ^1^Department of Pediatrics, Umm Al Qura University, Makkah, Saudi Arabia; ^2^Department of Pediatrics, International Medical Center, Jeddah, Saudi Arabia; ^3^Women's Health Center, International Medical Center, Jeddah, Saudi Arabia; ^4^Department of Pediatrics (Neonatal Division), McMaster University Medical Center, Hamilton, ON, Canada

## Abstract

Pfeiffer syndrome (PS) is an autosomal dominant disorder with three subtypes stemming from heterozygous mutations in the fibroblast growth factors FGFR1 and FGFR2. The subtypes overlap with heterogeneous clinical manifestations and variable prognosis dependent on neurological and respiratory compromise that impact short- and long-term outcomes and survival. We present a male, term infant with type II PS that was diagnostically suspected antenatally based on three-dimensional ultrasonographic findings that were confirmed postnatally by craniofacial tomography and magnetic resonance imaging. A new generation sequencing panel identified a unique *de novo* FGFR2, c.335 A > *G p*. Tyr112Cys variant, the first of its kind, and features that closely aligned with subtype II PS. Initial molecular results categorized the mutation as nonpathogenic, but it was later reclassified as pathogenic. Antenatal, multidisciplinary parental counseling about the tentative diagnosis and prognosis facilitated postnatal decisions that culminated in an informed choice for palliative care and early demise.

## 1. Introduction

Pfeiffer syndrome (PS; acrocephalosyndactyly Type V) is a rare genetic disorder characterized by premature closure of skull sutures (craniosynostosis), midfacial hypoplasia, ocular hypertelorism, brachydactyly, partial syndactyly of the fingers and toes, and abnormally broad and medially deviated thumbs and great toes [1–3]. Genetic analysis has linked the phenotype to different mutations of the fibroblast growth factor receptor 1 gene (FRGF1) on exon 8 and the FRGF2 gene on exon 10 [4]. The incidence of PS is estimated to be 1 : 100,000 live births [5, 6].

PS is categorized into three subtypes of which subtype 1 is recognized as relatively mild, with increasing degrees of phenotypic severity seen in type 2 and intermediate clinical manifestations in type 3 [7–10]. Type 2 PS is usually associated with a cloverleaf skull deformity, varying degrees of proptosis, and severe respiratory and neurological compromise. PS is usually detected in the newborn period or later, and few prenatal ultrasound diagnoses have been reported [5, 11, 12]. Severe cases of type II and III PS may develop life threatening complications in infancy and hence antenatal diagnosis is critical. Differential diagnosis includes other syndromic craniosynostosis. Advances in three-dimensional antenatal ultrasonography and magnetic resonance imaging have enabled earlier intrauterine diagnosis and parental counseling on short- and long-term outcomes [5, 11–13]. Linkage and mutational analyses have provided a better understanding of the pathogenesis of craniosynostosis and in some cases new findings have led to a change of the initial clinical diagnosis.

## 2. Case Presentation

We report on an antenatal suspected case of Pfeiffer type II syndrome. The c.335 A > *G p*. Tyr112Cys variant detected postnatally was a missense mutation reclassified from “likely pathogenic” to Class 1 “pathogenic”.

**Figure 1:**
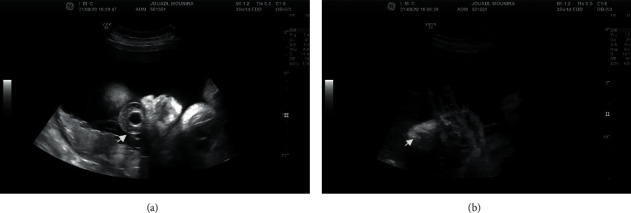
(a) Arrow indicates subluxation and proptosis of the right eye. (b) Arrow indicates deviated broad thumb.

A newborn male child was spontaneously conceived after eleven years of primary infertility. He was born to a 35-year-old primigravida without previous abortions and a 47-year-old father. Initial blood work, dating of the pregnancy, nuchal translucency, and scan for fetal anomalies at 20 weeks gestation were normal. The mother was referred to the fetal medicine department at 33 weeks gestation for an abnormal shape of the fetal head detected on ultrasound at a subsequent visit. The scan suggested premature closure of the cranial sutures resulting in a cloverleaf skull. There was dilatation of the anterior ventricle and the fetal forehead appeared high and broad. Proptosis of the eyes was evident and more severe on the right side (Figure 1(a)). The long bones appeared short, with proximal rhizomelia, while the thumbs and greater toes were wide and rotated (Figure 1(b)).

There was polyhydramnios with an amniotic fluid index of 24 cm and upper airway obstruction was also noted. A provisional diagnosis of PS type II was entertained, and the couple were appropriately counseled regarding a guarded, poor prognosis for the baby. The parents declined amniocentesis in view of the advanced stage of pregnancy and considered palliative care for the baby after birth.

**Figure 2:**
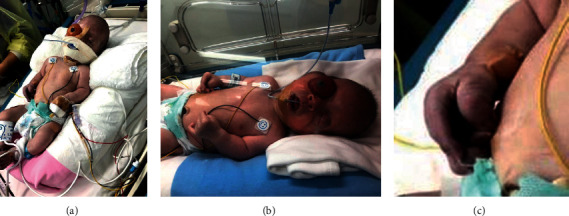
(a) Oropharyngeal airway to correct airway obstruction, long bones with proximal rhizomelia, and severe right eye proptosis. (b) Mid-facial hypoplasia, brachycephaly, cloverleaf skull, and bilateral ocular proptosis more severe on the right with absent eyelids (c) Broad thumb with medial deviation of the fingers.

The antenatal findings were clinically confirmed after birth Figure 2(a)–2(c) following elective delivery by Caesarian section at 37 weeks of gestation, due to breech presentation and a history of previous abdominal uterine myomectomy in the mother.

The baby had low-set and posteriorly rotated ears and a tense fontanel. Severe respiratory distress was due to upper airway obstruction secondary to macroglossia relative to the small oral cavity. The feet were clubbed bilaterally, without syndactyly of the fingers and toes. There was marked hypospadias. The orbits were shallow (Figure 2(b)) without the right upper and lower eyelids. There was profound right eyeball proptosis with severe hyperemic, chemotic conjunctiva, and an irregular cornea with a collapsed anterior chamber compared to the exorbitism of the left eye, which was displaced inferolaterally and displayed exposure keratopathy and lagopthalmus (Figure 2(a) and 2(b)).

**Figure 3:**
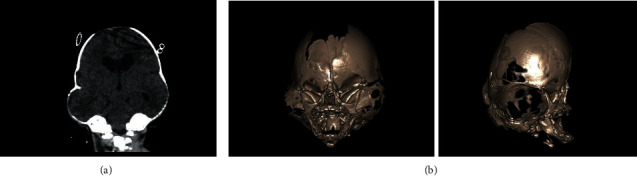
(a) CT scan of the head showing marked cloverleaf deformity with lateral ventricle dilatation. (b) Three-dimensional craniofacial reconstruct computed tomography of the skull (AP and lateral) demonstrating early closure and overlapping of all the cranial sutures with cloverleaf deformation.

Cranial ultrasound, CT scan, (Figure 3(a)) and magnetic resonance imaging of the brain revealed symmetrical lateral ventricular dilatation (anterior horns measured 16 mm in maximum transverse dimension) with no arterio-venous malformations and normal myelination of the white matter in both cerebral hemispheres. Abdominal ultrasound findings and cardiovascular examination were normal. A three-dimensional craniofacial reconstruction computed tomography of the skull (Figure 3(b)) was performed and substantiated the cloverleaf deformity with multiple craniosynostoses resulting in acrocephaly and brachycephaly.

The family were informed that cranial expansion with skull remodelling was urgently needed to decompress the raised intracranial pressure but that the surgical procedure was associated with significant risk. An electroretinogram was requested on both eyes with a tentative procedural plan for right unilateral orbital exenteration or enucleation. After careful consideration, the parents opted for comfort and palliative care and the baby died at 32 days of age and an autopsy was denied.

### 2.1. Genetic Analysis/Methods

The infant had a normal 46, XY karyotype. Whole exome sequencing CentoXome@ solo, including next-generation sequencing (NGS-based Copy Number Variation analysis) was done. Double stranded DNA capture baits against approximately 36.5 Mb of the human coding exome (targeting >98% of the coding RefSeq from the human genome build GRCh37/hg19) were employed to enrich target regions from fragmented genomic DNA with the Twist Human Core Exome Plus kit. The generated library was sequenced on an Illumina platform to obtain a minimum of 20-fold coverage depth for >98% of the targeted bases. A bioinformatics pipeline that read alignment to GRCh37/hg19 genome assembly, variant calling, annotation, and comprehensive variant filtering was applied. All variants with minor allele frequency (MAF) of less than 1% in gnomAD database, and disease-causing variants reported in HGMD®, in ClinVar or in CentoMD® were considered. The investigation for relevant variants focused on coding exons and flanking±20 intronic nucleotides of genes with clear gene-phenotype evidence (based on OMIM® information). All potential modes of inheritance patterns were considered, and the family history and clinical information were used to evaluate identified variants with respect to their pathogenicity and causality.

A mutation c.335 A > *G p*. Tyr112Cys was confirmed and is the result of an amino acid change from Tyr to Cys at position 112. According to the Human Gene Mutation Database (HGMD®; Professional. 2020.1), this variant was previously described as causing Pfeiffer syndrome [14–17]. ClinVar reports this variant as pathogenic (clinical testing, Variation ID: 449398) [18], and it is classified as likely pathogenic Class (2) according to the American College of Medical Genetics recommendations [19]. The initial interpretation was that this was a variant in a heterozygous state for this proband. Variants in this gene are associated with autosomal dominant disorders with the phenotypic spectrum of the FGFR2 gene (OMIM®: 176943). HGMD and Mutation Taster reported this variant as disease-causing for PS type 2 (PubMed Unique Identifier: 27683237, 10394936). In the heterozygous state this variant is absent from the control population. The variant was not detected in the parents, and therefore it was determined to have a *de novo* origin.

Based on new evidence for the FGFR2 gene from the National Center for Biotechnology Information reference sequence NM_001320654.1 variant, c.504-8G > A *p*. Tyr112Cys, and NM_001383.3 transcript reference sequence for c.335 A > *G p*. Tyr112Cys, the missense variant in this infant was reclassified from likely pathogenic to pathogenic [20]. The genetic diagnosis of an autosomal dominant disorder with the phenotypic spectrum of the FGFR2 gene was therefore confirmed.  Table 1 outlines all the *de novo* mutations reported in PS type 2 from January 1^st^, 2000, up to the present [5, 11, 16, 21–23].

## 3. Discussion

Pfeiffer syndrome is an autosomal dominant disorder caused by mutations in the FGFR1 and FGFR2 genes. Prenatal diagnosis of Pfeiffer syndrome is challenging and a literature search by Giancotti et al. identified a total of 18 case reports or case series [5]. In a 5-year, multicenter retrospective study, among 41 cases of craniosynostosis, 73% (*n* = 30) were syndromic of which 15 were identified with PS [12]. Twelve cases were detected prenatally and a cloverleaf skull was found in nine fetuses which led to a diagnostic suspicion of PS [12]. The low incidence and the wide variability of morphological findings in PS, which can also be related to other nonsyndromic craniosynostoses and chromosomal deletion disorders, make it difficult to suspect this syndrome in early pregnancy [12]. Although most cases of PS are diagnosed in the neonatal period, prenatal diagnosis is possible. Three-dimensional obstetric ultrasound is the first-line diagnostic tool for suspected PS, being useful to verify suture closure in the second and third trimesters of pregnancy for the most severe cases [21, 24, 25]. Our patient had the Pfeiffer syndrome type 2 phenotype with the cloverleaf skull which is present in greater than 50% of the cases, is a consequence of premature fusion of all sutures which may occur as early as 23 weeks gestation [5, 12, 26]. However, the cranial asymmetry while characteristic of the Type II phenotype, may also occur as part of the Beare–Stevenson syndrome with FGFR2 mutation albeit with less cranial deformation [12, 27, 28].

Our patient presented with hypertelorism and severe ocular proptosis, characteristics that are similar to the PS type II cases described in the literature with de novo mutations in the FGFR2 gene [5, 11]. In the case series of overall PS described by Giancotti et al., 44.4% of the patients had the same features, but pronounced ocular proptosis occurred more frequently in association with the FGFR2 gene mutation [5]. This condition can lead to endophthalmitis and rupture of the eyeball, so periodic evaluation by the ophthalmologist is necessary. Corrective surgery aims at decompression of the brain and remodeling of the skull, elongation and expansion of the bony orbits to accommodate the globes and enable eyelid closure, and unblocking the compromised nasopharyngeal airways by advancement of the naso-maxillaryzygomatic complex [6]. Congenital upper airway anomalies related to midface hypoplasia and macroglossia as in our case may cause chronic hypoventilation and hypoxia leading to neurodevelopmental deficits and mortality [5].

Among the 32 cases of PS diagnosed prenatally in the reported literature [5, 11], ten (31%) were attributed to mutations in the FGFR2 and two in the FGFR1 gene [5]. Harada et al. [12] reported on 15 unique cases of PS diagnosed prenatally but failed to denote the relevant references pertaining to the cases which may have overlapped the case series documented by Giancotti et al. [5]. The more severe types of Pfeiffer Syndrome are due to de novo mutations, but the presence of mosaicism in one of the parents must be investigated. Familial recurrence risk should be addressed within the scope of genetic counseling; however, most Type II cases are nonfamilial [7, 29]. Chokdeemboon et al. reported that in 12 sporadic cases of PS in Thai individuals, 50% were associated with advanced paternal age [30]. Glaser et al. screened 11 families with PS and prove at a molecular level that all the FGFR2 mutations had a paternal origin [31]. Advanced paternal age was noted for the fathers of patients with Crouzon syndrome or Pfeiffer syndrome, compared with the fathers of control individuals (34.50 ± 7.65 years vs. 30.45 ± 1.28 years, *P* < .01). It is well established that paternal compared to maternal age has a greater impact on cases of sporadic autosomal dominant congenital disorders such as Apert, Crouzon, Pfeiffer, Noonan, and Costello syndromes, multiple endocrine neoplasia (types 2A and 2B) and achondroplasia [32]. The main cause is the difference in gametogenesis between men and women. Female oocytes do not go through DNA replication at a mature age in contrast to male spermatogenesis. Ageing leads to more DNA replications during spermatogenesis in testicles and increases the risk of copy error mutations such as small deletions and insertions [33]. It is well-established that paternal age has a greater impact on cases of sporadic autosomal dominant disorders including PS [33, 34]. Prenatal diagnosis of PS allows for early referral to tertiary centers, timely genetic counseling, and close follow-up and intervention planning in the prenatal and postnatal stages.

Craniosynostosis syndromes are associated with several gene variants including gain-of-function mutations of the FGRF 1–3 genes. Studies show different degrees of overlap across the spectrum of syndromic, nonsyndromic, and chromosomal disorders. The newly discovered variants are likely to enhance our understanding of the underlying pathology. New-generation sequencing panels for molecular gene analysis can elucidate the presence of pathogenic versus nonpathogenic variants and uniform classification using up-to-date guidelines [19, 35] helps better clinical management and parental counseling with appropriate interdisciplinary decision making and intervention in such cases.

## Figures and Tables

**Table 1:** De novo mutation reports of Pfeiffer syndrome type 2 involving the FGFR2 gene.

Author/year	Inheritance	Sex	Craniofacial anomalies	Limb/digital anomalies	Outcome	Mutation
Benacerraf et al./2000 [21]	de novo mutation	M	Severe acrocephalic skull, fused sutures, flat facies, frontal bossing	Syndactyly of digits of both hands and feet, wide hallux	Still born	Mutation from G to T at codon 314 leading to alanine to serine amino acid substitution in axon 9 of the gene
Blaumeiser et al./2004 [22]	de novo mutation	M	Cloverleaf skull, flattening of the midface, flat and broad nasal bridge, bilateral ocular proptosis, short neck	Prominent thumbs and great toes, micropenis	Stillborn	1019A > *G* (Y340 C)
Gomez et al./2013 [23]	Heterozygosity	N/A	Cloverleaf cranium, severe proptosis	No observed hand and foot deformities	Pregnancy terminated	c.870 >T p.Trp 290Cys. Presumed de novo
Ohishi et al./2016 (case 5) [16]	Sporadic-de novo	M	Cloverleaf, exophthalmos choanal atresia	Humeroradial synostosis, broad 1^st^ toes	Developmental delay	c.870 G > T p.Trp290Cys
Ohishi et al./2016 (case 6) [16]	Sporadic-de novo	M	Brachycephaly, cloverleaf, exophthalmos, high arched palate	Radioulnar fusion, radially deviated thumbs, broad 1^st^ toes	Developmental delay	c.870 G > T p.Trp290Cys
Ohishi et al./2016 (case 7) [16]	Sporadic-de novo	M	Brachycephaly, cloverleaf,low-set ears, exophthalmos, saddle nose	Humeroradial synostosis, broad 1^st^ toes	Developmental delay	c.870 G > T p.Trp290Cys
Ohishi et al./2016 (case 8) [16]	Sporadic-de novo	F	Cloverleaf, exophthalmos	Humeroradial synostosis, broad first toes, syndactyly of 1st and 2nd toes	Developmental delay	c.1019 A > *G* p.Trp340Cys
Giancotti et al./2017 [5]	de novo mutation.	N/A	Cloverleaf, dolichocephaly, frontal bossing, depressed nasal bridge, proptosis, severe hypertelorism, upper jaw hypoplasia	Short humerus, 5^th^ finger clinodactyly, many superimposed phalanges. In lower limbs, shorter bones than normal, curved right tibia, clubfeet	Stillborn	c.870G4T(p.Trp290Cys) mutation in exon 7
Torres-canchala et al./2020 [11]	Sporadic-de novo	F	Cloverleaf-shaped skull, facial hypoplasia, low ears, exophthalmos	Wide, broad, and deviated thumbs and hallux.	Psychomotor retardation, sleep apnea	c.940–1G > C

## Data Availability

Data supporting the findings of this study are available upon request from the corresponding author.
